# Locked Twins in a Primigravida: A Rare Obstetric Emergency Successfully Managed by Cesarean Section—A Case Report

**DOI:** 10.1155/crog/2974129

**Published:** 2026-04-24

**Authors:** Getahun Wodalo, Amanuel Admasu Yayna, Amdetsiyon Yemane, Adane Ayza, Ashebir Tesfaye, Bereket Worana, Belachew Shote, Biranu Mamo, Elsabeth Samuel, Getu Kassaye, Wokil Wolde Dana

**Affiliations:** ^1^ Department of Gynecology and Obstetrics, Wolaita Sodo University Comprehensive and Specialized Hospital, Wolaita Sodo, South Region, Ethiopia; ^2^ Department of Gynecology and Obstetrics, Debre Berhan University, Asrat Woldeyes Health Science Campus, Debre Berhan, Amhara Region, Ethiopia, dbu.edu.et

**Keywords:** case report, chin-to-neck locking, Ethiopia, locked twins, twin pregnancy

## Abstract

**Background and Aims:**

Locked twins are a rare and life‐threatening obstetric complication, occurring in approximately 1 in 1000 twin pregnancies, most commonly during labor when the first twin presents as breech and the second as cephalic. This condition poses significant risks to both the mother and fetuses if not promptly recognized and managed. We report a rare case of chin‐to‐neck locked twins and highlight the importance of early diagnosis and meticulous intraoperative management.

**Methods:**

A 20‐year‐old primigravida at 9 months of amenorrhea presented with pushing down pain for 12 h and ruptured membranes for 4 h. Clinical examination revealed a twin pregnancy with the first twin in breech presentation and a fetal heart rate of 98–100 beats per minute, while the second twin was in cephalic presentation with a fetal heart rate of 136–138 beats per minute. An emergency cesarean section was performed. Intraoperatively, a chin‐to‐neck interlocking of the twins was identified. Both twins were successfully delivered through the lower segment transverse uterine incision and careful intraoperative disimpaction without intraoperative complications.

**Results:**

The first twin was a male weighing 1600 g with Apgar scores of 5 and 7 at 1 and 5 min, respectively, and died 1 h postpartum due to respiratory failure related to prematurity and with a possible contribution from intrapartum hypoxia associated with interlocking of the twins. The second twin was a female weighing 1800 g with Apgar scores of 7 and 9, who required brief neonatal intensive care for respiratory distress syndrome and subsequently recovered well. The mother had an uneventful postoperative course.

**Conclusion:**

This case underscores the critical importance of early recognition of locked twins, timely surgical intervention, and careful intraoperative techniques to minimize maternal and fetal morbidity and mortality. Vigilant antenatal surveillance and preparedness in high‐risk twin pregnancies are essential to prevent catastrophic outcomes associated with this rare obstetric emergency.

## 1. Introduction

Twin pregnancy is a high‐risk obstetric condition associated with increased maternal and perinatal morbidity and mortality, particularly in low‐ and middle‐income countries. In Ethiopia, perinatal care for twin pregnancies is mainly provided through routine antenatal care services, with ultrasonography increasingly available in urban and tertiary centers but still limited in many peripheral and rural facilities. As a result, antepartum detection of twin pregnancies is incomplete; studies from central Ethiopia report that approximately 60%–70% of twin pregnancies are diagnosed before labor, while a substantial proportion are identified intrapartum (1).

Interlocking of twins commonly occurs when the after‐coming head of the first fetus, usually in breech presentation, is locked with the head of the second cephalic presenting fetus. There are different etiological factors for the interlocking of twins, of which the most important are the age and parity of the mother and the size of the twins. Interlocking twins are more common in primigravida as basal uterine tone may be greater in the first pregnancy resulting in stronger uterine contractions during labor (2). This complication of twin delivery happens rarely—1 in every 90,000 deliveries or 1 in every 1000 twin deliveries (3). The stillbirth rate of locked twins is estimated to be 50% (2, 4).

In our case, the twin pregnancy was diagnosed intrapartum after the onset of labor, reflecting the ongoing challenges of early detection of multiple gestations in resource‐limited settings like Ethiopia and Eastern Africa and associated increased risk of adverse perinatal outcomes. This case is reported to highlight the diagnostic and management challenges of locked twins in a low‐resource setting.

## 2. Case Summary

A 20‐year‐old primigravida woman presented with complaints of pushing down pain for the past 12 h and a gush of fluid from the vagina for the last 4 h. She was unsure of the exact date of her last normal menstrual period but reported being amenorrheic for 9 months. She had not undergone any early ultrasound. With these complaints, she visited a nearby health center and was referred to our hospital for a diagnosis of twin pregnancy with the first twin in breech presentation for better care. She had no history of vaginal bleeding, fever, or any obstetric complications other than the current complaints and no underlying medical conditions. Her antenatal care visits were at a nearby health center, and her medical, surgical, psychosocial, and family histories were unremarkable.

Upon physical examination, her vital signs were within normal ranges: blood pressure 110/70 mmHg, pulse rate 92 beats per minute, temperature 36.8°C, and respiratory rate 18 breaths per minute. Her conjunctiva was pink. On abdominal and pelvic examination, a distended gravid uterus with four strong uterine contractions per 10 min, without clinical evidence of increased uterine tone, the fetal heart rates of the first and second twins were 98–100 and 136–138 beats per minute, respectively. The cervix was fully dilated, and the first twin was in complete breech presentation at station +1, with ruptured membranes and clear amniotic fluid.

With a diagnosis of second‐stage labor with a twin pregnancy and the first twin in breech presentation, an obstetric ultrasound revealed the first twin in breech presentation, weighing approximately 1700 g, and the second twin in cephalic presentation, weighing approximately 1800 g. An emergency cesarean delivery was decided upon, and after informed consent was obtained, the mother was taken to the operating room. A lower transverse incision was made, and upon entry into the uterine cavity, interlocking of the twins was detected. The chin of the second twin was tightly impinged on the right side of the neck of the first twin above the pelvic inlet. The interlocking of the twins was resolved by gently disimpacting the chin of the second twin from the neck of the first twin, followed by gently pushing the second twin′s head toward the fundus to relieve the interlocking. The first twin was then delivered by pulling on the lower extremities, followed by the delivery of the second twin.

Both twins were successfully delivered through the lower segment transverse uterine incision and careful intraoperative disimpaction without requiring additional surgical procedures and without intraoperative complications. The first twin, a male, weighed 1600 g and had Apgar scores of 5 and 7 at 1 and 5 min, respectively. There was a 2 × 2 cm abrasion on the right side of the neck where the chin of the second twin had impinged. After resuscitation, the first twin was transferred to the neonatal intensive care unit and placed on mechanical ventilation in CPAP mode. Despite neonatal resuscitation and ventilatory support, the first twin died 1 h postpartum due to respiratory failure related to prematurity and low birth weight with a possible contribution from intrapartum hypoxia associated with interlocking of the twins. Umbilical cord blood gas analysis and complete neonatal postmortem examination were not available.

The second twin, a female, weighed 1800 g and had Apgar scores of 7 and 9 at 1 and 5 min, respectively. She was transferred to the neonatal care unit with a diagnosis of respiratory distress syndrome and low birth weight. She was placed on intranasal oxygen and received antibiotics, which were discontinued after 48 h when the blood culture results were negative for bacterial growth.

The mother was discharged with the surviving baby after a full recovery and 4 days of postnatal care. On follow‐up after 1 week, her wound had healed well, and no complications were detected.

## 3. Discussion

Locked twins represent a rare but severe intrapartum complication that occurs in approximately 1 in 1000 twin pregnancies associated with high perinatal mortality, particularly when diagnosis occurs late in labor (2). Predisposing factors for interlocking twins typically include small babies, a large pelvis, primigravidity, oligohydramnios, uterine hypertonicity, early rupture of the second sac, and monochorionic monoamniotic twins. When the babies are larger, they tend to lock above the pelvic inlet, while smaller twins may lock after descending into the pelvis (3). Due to the difficulty in disimpacting the heads, which can result in the death of one or both twins, the perinatal mortality rate is high, exceeding 40% (5).

Our case is unique because the diagnosis of interlocking was made intraoperatively and delivery was achieved with a lower uterine segment transverse incision without extension. An ulcer was noted on the first twin at the site of tight interlocking. The difficulty in disengaging the two heads was resolved by making a wide uterine incision on the lower segment and gently pushing the head of the second twin, allowing safe dislodging of the interlocking during the operation. Delivery of both twins was achieved because interlocking occurred above the pelvic brim and abdominal delivery was performed without vaginal manipulation, thereby avoiding additional fetal or maternal trauma.

Nissen classified twin entanglement into four distinct types. The first type, collision, occurs when contact between any part of one baby and its co‐twin prevents the engagement of either twin. The second type, impaction, involves part of one twin impacting the surface of its co‐twin, leading to only partial engagement of both simultaneously. The third type, compaction, is when the leading fetal parts of both twins fully engage at the same time, filling the pelvic cavity and preventing descent or disengagement of either twin. Finally, interlocking happens when the chin of one twin is in intimate contact with that of its co‐twin, either above or below the pelvic brim. Our case aligns with the collision type according to Nissen′s classification (6).

Interlocking above the pelvic brim, as seen in our case, generally does not pose a severe threat to both twins, and abdominal delivery should be prioritized without any vaginal manipulation. The successful delivery of live babies in this instance can be attributed to the interlocking occurring above the pelvic brim, despite the wound observed at the interlocking site on the first twin and the subsequent postnatal death. However, when interlocking occurs at or below the pelvic brim, it becomes a serious complication, often leading to the death of the first twin or, in some cases, both. In such scenarios, there are reports where successful vaginal delivery was achieved by decapitating the first twin when it was already deceased (7). There are also reports indicating the use of anesthetic drugs to relax uterine tone, allowing for the successful application of the Zavanelli maneuver to unlock the twins. This approach can facilitate the vaginal delivery of both twins without the need for destructive procedures to the first twin (8). Some other report describes failure of manual maneuvers to deliver vaginally (2). However, these approaches require early diagnosis, skilled personnel, and adequate anesthesia support, which may not be readily available in many low‐resource settings.

This case highlights the particular challenges faced in low‐income countries, where routine ultrasonography before or at the onset of labor is not universally available and twin pregnancies may remain undiagnosed until late in labor or even during surgery. Late diagnosis of locked twins, as demonstrated in this case, significantly limits management options and may contribute to adverse perinatal outcomes despite timely emergency cesarean delivery. Our report therefore underscores the importance of improving antenatal detection of twin pregnancies, strengthening intrapartum surveillance, and ensuring prompt referral to facilities capable of advanced obstetric and neonatal care in resource‐limited settings.

## 4. Conclusion

Locked twins, though rare, may present as a severe obstetric emergency when twin pregnancy is not diagnosed antenatally.

The management of locked twins should be individualized, with an emphasis on anticipation and prevention. Proper prenatal care, including sonography and thorough evaluation, is essential. High‐risk mothers should be identified and managed in facilities equipped with the necessary tools and skilled personnel. Even in high‐income settings, early preterm rupture of membranes and onset of labor in twin pregnancies require urgent evaluation and rapid admission to prevent complications. Elective abdominal delivery should be considered for cases where the first twin is in a noncephalic presentation to avoid the potentially catastrophic complication of locked twins. In cases where locking occurs, swift and appropriate actions must be taken to minimize maternal and fetal morbidity and mortality. Timely recognition of potential cases and punctual cesarean sections can significantly reduce adverse outcomes.

**Patient Perspective:**

“At first, when I started to have labor pain, it was the normal one I had been informed of during my antenatal visits. But with time, as this pain grew stronger and lasted for hours, I knew something was not right. When my water broke, I went to the nearby health center. A midwife examined me there and told me that I was carrying twins, which I had not known in advance because I have never been in the setup with ultrasound. This was a shock and quite bothering, all the more so since one of them was in breech position. I was referred to the bigger hospital for better care.

There, the doctors informed me that I had to go for a cesarean section so that the babies could be born safely. Well, I was terrified but simply trusted the doctors. After the surgery, the doctors told me that my twins were ‘locked’ inside me and that is the reason behind such difficult labor. It came as a relief when both the babies were born alive, but soon after, I was made to understand that my son did not survive. That was tough to bear. I felt thankful that my daughter made it, and the doctors assured me she was doing well after a short stay in the NICU.

The post‐operative care by the doctors and nurses was excellent, and I was able to recover well from the surgery. Of course, emotionally, it was a difficult time for me as I mourned the loss of one baby while simultaneously experiencing the joy of my remaining child. I am grateful to the team at this hospital for saving my and my daughter′s lives. I hope that other women who experience twin pregnancies get the same type of attention and care for their conditions, and that they do not have to go through such complications.”

## Author Contributions

Dr. Amanuel Admasu Yayna had full access to all of the data in this study and takes complete responsibility for the integrity of the data and the accuracy of the data analysis. Dr. Amanuel Admasu Yayna affirms that this manuscript is an honest, accurate, and transparent account of the study being reported, that no important aspects of the study have been omitted, and that any discrepancies from the study as planned (and, if relevant, registered) have been explained. All authors are involved in the diagnosis and treatment including the operation, wrote the case report, and contributed to data analysis and the drafting or revising of the article.

## Funding

No funding was received for this manuscript.

## Disclosure

All authors have read and approved the final version of the manuscript.

## Consent

Informed written consent has been obtained from the patient for all case details to be reported in the journal.

## Conflicts of Interest

The authors declare no conflicts of interest.

## Data Availability

No new datasets were generated for this case report. All relevant clinical information is included within the article. Additional data that support the findings of this case are available from the corresponding author, Dr. Amanuel Admasu Yayna, upon reasonable request. The data are not publicly available to protect patient privacy and confidentiality.
